# Molecular characterization and overexpression of *mnp6* and *vp3* from *Pleurotus ostreatus* revealed their involvement in biodegradation of cotton stalk lignin

**DOI:** 10.1242/bio.036483

**Published:** 2018-12-24

**Authors:** Yan Wang, Guoqing Li, Xiaoyu Jiao, Xi Cheng, Muhammad Abdullah, Dahui Li, Yi Lin, Yongping Cai, Fan Nie

**Affiliations:** 1 School of Life Sciences, Anhui Agricultural University, Hefei 230036, China; 2 Horticultural Research Institute, Anhui Academy of Agricultural Sciences, Hefei 230031, China

**Keywords:** *Pleurotus ostreatus*, Class II peroxidase, Bioinformatics, Overexpression, Lignin degradation

## Abstract

Fungal secretory heme peroxidase (Class II POD) plays a significant role in biomass conversion due to its lignin-degrading activity. In this study, genome-wide identification and bioinformatics were performed to analyze *Pleurotus* *ostreatus* peroxidases (*PoPOD*s). A total of six manganese peroxidases (MnPs) and three versatile peroxidases (VPs) were obtained. Bioinformatics analysis and qRT-PCR showed that *P. ostreatus mnp6* (*Pomnp6*) and *P. ostreatus vp3* (*Povp3*) could be involved in lignin degradation. Both *Pomnp6* and *Povp3* transgenetic fungi showed significantly increased lignin degradation of cotton stalks. ^1^H-NMR revealed that *Pomnp6* and *Povp3* may preferentially degrade S-lignin in cotton stalks and mainly break β-*O*-4′ bond linkages and hydroxyl. These results support the possible utility of *Pomnp6* and *Povp3* in natural straw resources and development of sustainable energy.

## Abstract (precis)

**Summary:** *Pleurotus ostreatus mnp6* and *vp3* could degrade cotton stalk lignin and may preferentially degrade S-lignin.

## INTRODUCTION

The main carbohydrates in crop straw are lignin, cellulose and hemicellulose. Lignin is a heterogeneous aromatic polymer; the major C_6_–C_3_ (phenylpropanoid) units of lignin are connected by ether and carbon-carbon linkages, such as β-*O*-4′, 4-*O*-5′, β-β′, β-1′, β-5′ and 5-5′ (Kumar et al., 2009). The bio-conversion of plant lignocellulose to glucose is an important part of second generation biofuel production. However, due to the complex and heterogeneous structures of lignin, straw resources are difficult to fully utilize (Lan et al., 2011). Previous research has shown that white-rot fungi could break down lignin polymers with their extracellular oxidases, indicating great significance for the application of biomass conversion (Bugg et al., 2011).

Lignin-degrading enzymes consist of laccase (Lac, EC1.10.3.2), lignin peroxidase (LiP, EC1.11.1.14), manganese peroxidase (MnP, EC1.11.1.13) and versatile peroxidase (VP, EC1.11.1.16) (Isroi et al., 2011). Among them are ligninolytic class II secreted heme peroxidases (PODs) which include lignin peroxidase, manganese peroxidase and versatile peroxidase (Manavalan et al., 2015). Manganese peroxidase, a heme-containing enzyme protein, in the presence of H_2_O_2_ and Mn^2+^, can oxidize aromatic compounds (Pollegioni et al., 2015). Versatile peroxidase, which has the enzyme activity of both LiP and MnP, can oxidize Mn^2+^, veratryl alcohol (VA) and 2,6-dimethoxy phenol (DMP) etc., playing an important role in lignin biodegradation (Martínez et al., 1996; Ruiz-Duenas et al., 2009).

The heterologous expression of MnP and VP enzymes from various white-rot fungi have been widely studied (Wang et al., 2018; Campos et al., 2016). Furthermore, numerous studies have been conducted on the functions of fungal Lacs, MnPs and VPs, including *Gloeophyllum trabeum lcc3* (*Gtlcc3*) for lignin degradation in Japanese cedar wood-containing medium (Arimoto et al., 2015) and the *lac1* from *Polyporus brumalis* for significant contribution to the increased sugar yields (Ryu et al., 2013).

*Pleurotus ostreatus* is an important edible-fungi and is widely cultivated in China (Qu et al., 2016). In addition to its edible value, *P. ostreatus* also exhibits a strong ability to degrade lignin. *P. ostreatus* can produce Lacs, MnPs and VPs, but not LiPs (Suetomi et al., 2015). In a previous study, *P. ostreatus vp1* (*Povp1*) and *Povp2* have been proven to have the function of degrading non-phenolic lignin dimers and lignin polymers, respectively (Fernández-Fueyo et al., 2014; Salame et al., 2014). Then, the secretome of the model white-rot agaric *P. ostreatus* growing on woody (poplar wood) and non-woody (wheat straw) lignocellulose was analyzed and compared with that from a glucose medium (hat), the result showed that Mnp3, Mnp6 and VP1 were overproduced on straw and poplar, but not found in hat, while VP2 was only found on straw and VP3 was only found on poplar (Fernández-Fueyo et al., 2016). However, there was no specific experimental evidence for whether *Pomnp3*, *Pomnp6* and *Povp3* have the function of lignin degradation. Recently, with the development of *Agrobacterium-mediated transformation* (*ATMT*) in fungal transgenetic research, the efficiency of genetic transformation has increased significantly compared to protoplast transformation by the PEG-CaCl_2_ method (Guo et al., 2016; Long et al., 2018)*.* This technology will enhance the excavation of many genes in *P. ostreatus*.

In the present study, we explored the biological significance of *P. ostreatus* Class II PODs using bioinformatics and molecular cloning. Two PODs, *Pomnp6* and *Povp3*, were selected from members of the POD family based on the above findings and bioinformatic analysis. Then *Pomnp6* and *Povp3* were overexpressed in fungi via *ATMT* to further validate their functions. The results supported the possible utility of the *Pomnp6* and *Povp3* in natural straw resources and development of sustainable energy.

## RESULTS

### Phylogenetic analysis of PoPODs

**Fig. 1.**
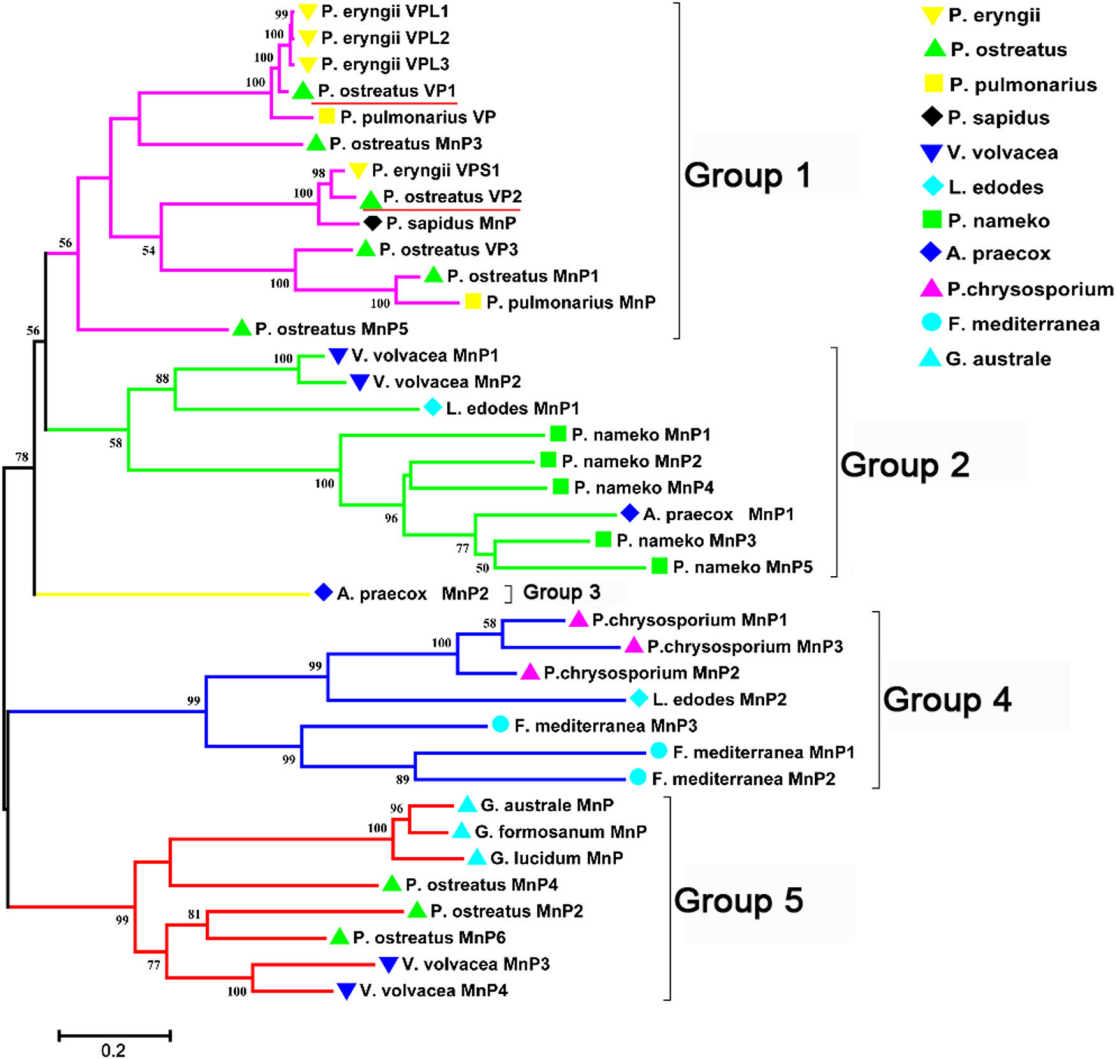
**Evolutionary analysis of basidiomycete PODs.** Nine PoPODs were identified and classified from the whole genome. Another 27 PoPODs were obtained from the National Center for Biotechnology Information (NCBI). This phylogenetic tree was constructed with MEGA5.1 software using the Maximum Likelihood method (bootstrap=1000). Green triangles represent the *P. ostreatus* POD gene family members. Bottom left bar represents evolutionary distance.

Nine putative peroxidase genes (*PoPOD*s) were obtained from the Joint Genome Institute (JGI) (http://genome.jgi.doe.gov/PleosPC15_2) and other basidiomycetes POD gene sequence data was obtained from National Center for Biotechnology Information (NCBI) (https://www.ncbi.nlm.nih.gov/) (Alfaro et al., 2016; Castanera et al., 2016; Riley et al., 2014). Then a phylogenetic tree of 36 PODs from basidiomycetes were constructed with MEGA5.1 using maximum likelihood method (gamma distributed with invariant sites, G+I) (bootstrap=1000). The result showed that these PODs were divided into five groups. PoPODs mainly existed in the groups 1 and 5 which were close to *Pleurotus pulmonarius*, *Pleurotus eryngii* and *Volvaria volvacea* (Fig. 1). Subsequently, gene structures analysis indicated that *Pomnp5*, *Povp2* and *Povp3* have similar gene structures (Fig. S1). Conserved motifs analysis showed that *Povp1* had distinctive feature in genetic characteristics and the motifs of other PoPODs were highly similar (Fig. S1). In addition, all PoPODs contained peroxidase domain and fungal peroxidase extension region based on the annotation of Pfam (Table S1).

### Lignin content and gene relative transcript abundance

In order to understand the degradation process of the cotton stalks after culturing *P. ostreatus*, the lignin content was determined at different stages of cultivation. The results showed that the change of lignin content mainly occurred in the growth process of *P. ostreatus* mycelium, when it was grown in fruiting stage, the lignin content remained unchanged. It showed that the degradation of cotton stalks lignin mainly occurred during the growth process of *P. ostreatus* mycelium (Fig. S2).

**Fig. 2.**
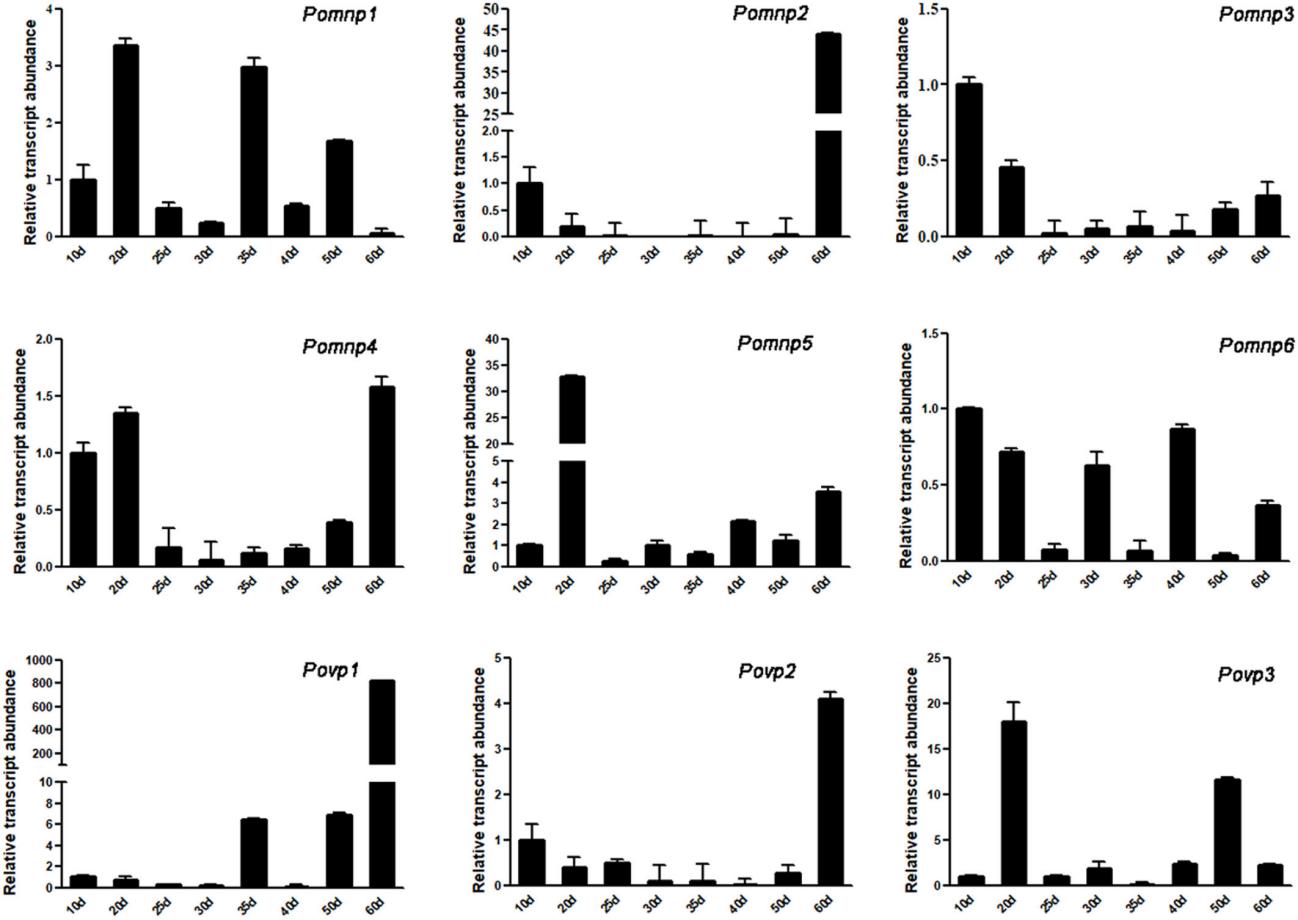
**Gene relative transcript abundance of *PoPOD*s in different periods.** *P. ostreatus* was cultured to the mycelium stage for 10 days, 20 days, 25 days and 30 days and cultured to fruiting stage for 35 days, 40 days, 50 days and 60 days. The Y-axis represents gene relative transcript abundance and the X-axis indicates incubation periods. The gene relative transcript abundance of 10 was set as ‘1’. Each sample set three duplications. Values are mean± s.d. (*n*=3).

In addition, the relative transcript abundance of *PoPOD*s was assayed (Fig. 2). The amplification efficiency of all genes is shown in Table S6. The qRT-PCR showed that these genes exhibited diverse transcript abundance patterns at various stages (Fig. 2). Except *Pomnp2* and *Povp1*, the transcript abundance of other seven genes was high in the mycelium and the transcript abundance of seven genes (*Pomnp1*, *Pomnp2*, *Pomnp4*, *Pomnp6*, *Povp1*, *Povp2* and *Povp3*) was high in the fruiting body stage.

The mRNA expression level of *Pomnp3* and *Pomnp5*, showing significant increase at mycelium stage and decrease at fruiting stage, were in accordance with the peak of lignin degradation (Fig. S2), suggesting that they may be associated with lignin degradation. *Pomnp2* transcript abundance was low in mycelium stage but increased significantly in fruiting stage, revealing it may be involved in the formation of the fruiting body. In addition, *Pomnp1*, *Pomnp4*, *Pomnp6*, *Povp2* and *Povp3* had high level of transcript abundance both during mycelium and fruiting stage, so they were inferred to be involved in lignin degradation and the formation of the fruiting body. Based on these data, we speculated that *Pomnp6* and *Povp3* may be associated with lignin degradation of cotton stalks. In the next section, we will further study the function of *Pomnp6* and *Povp3*.

### Overexpression of *Pomnp6* and *Povp3*

**Fig. 3.**
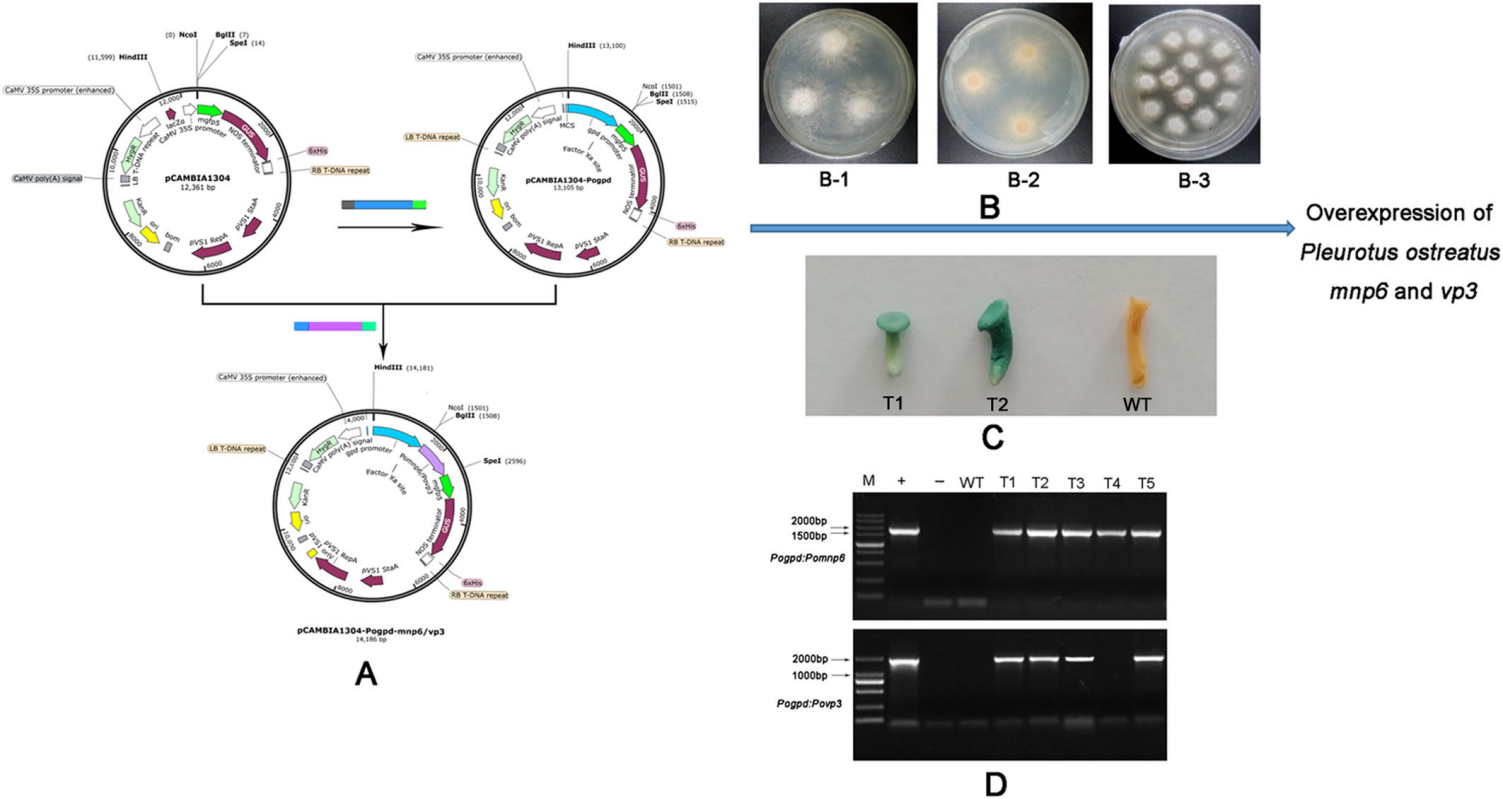
**Vector reconstruction and overexpression.** (A) *Pogpd* promoter was cloned and connected to the carrier to replace *CAMV35S* promoter in the pCAMBIA1304 vector. The target genes then were inserted into the expression plasmid pCAMBIA1304-*Pogpd*. *Hyg* (*hygromycin phosphotransferase* gene) was used as a selecting maker. *Gus* (*β-glucuronidase* gene) was used as a reporter gene. (B-1, B-2) AS (acetosyringone) induced hyphal growth (B-3) *P. ostreatus* transformed strain was selected by hygromycin; (C) GUS analysis (D) PCR analysis of *Pomnp6* and *Povp3* transformants. The PCR analysis used specific primers to amplify the 1800 bp internal fragment of *gus*. WT, wild type; T1–T5, genomic DNAs from putative transformants; +, positive control with plasmid pCAMBIA1304-*Pogpd*; –, negative control with pure water.

In order to investigate their function for lignin degradation, driven by the *gpd* promoter *Pomnp6* (GenBank accession no. MF681783) and *Povp3* (GenBank accession no. MF681784) were cloned into pCAMBIA1304 (Fig. 3A) and transformed into *P. ostreatus* using *ATMT* methods (Fig. 3B). Subsequently, five transformants were randomly selected to verify whether the reporter gene *β-glucuronidase* (*gus*) was inserted into *P. ostreatus* genome. The results showed that these transformants showed the presence of amplification products of *gus* (Fig. 3D). Additionally, a GUS histochemical assay showed that after 10 h of staining, the immature fruiting bodies of *P. ostreatus* transformants turned blue in the GUS-stained buff, whereas the wild type did not turn blue, indicating the activity of GUS in the transformants (Fig. 3C). The results showed that exogenous T-DNA has been successfully integrated into the genome of *P. ostreatus.*

### Gene-relative transcript abundance and lignin degradation rate of transformants

**Fig. 4.**
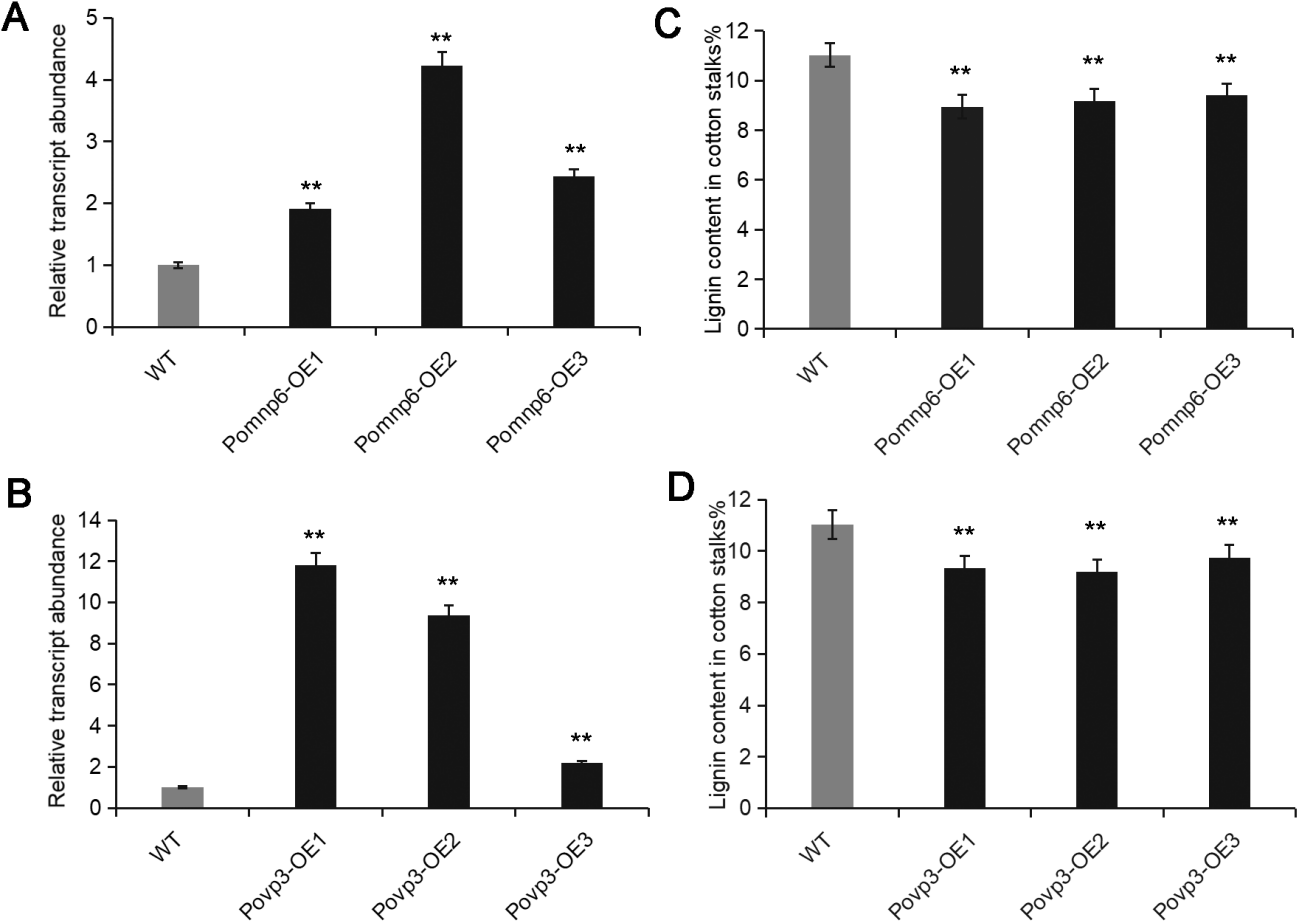
**Gene-relative transcript abundance and lignin degradation rate analysis of transformants.** (A,B) Gene relative transcript abundance of wild type and transformants after cultivating in the cotton solid medium for 20 days. (C,D) Lignin degradation rate of wild type and transformants after cultivating in the cotton solid medium for 30 days. Each sample had three duplications. Values are mean±s.d. (*n*=3). Student's *t*-test was used in this study. **P<0.01, *P<0.05.

After cultivating in the cotton solid medium for 20 days, the gene relative transcript abundance of *Pomnp6* and *Povp3* in the transformants were ∼2–4-fold and 2–11-fold higher than those in wild-type fungi, respectively (Fig. 4A,B).

To clarify the function of these two genes, cotton stalks were cultured for 30 days with wild-type and overexpressed *P.* *ostreatus*, and the lignin content of cotton stalks was determined. The lignin degradation rate of cotton stalks by wild-type strain was 48.56±7.04%. The lignin degradation rate of cotton stalks by transformants was 57.18±2.29% (*Pomnp6*) and 56.03±2.87% (*Povp3*), respectively. Compared with wild type, the lignin degradation rate of overexpressed strains was increased by 8.62% (*Pomnp6*) and 7.46% (*Povp3*), respectively (Fig. 4C,D). Therefore, it directly proved that *Pomnp6* and *Povp3* participated in the biodegradation of cotton stalks lignin. But how did *Pomnp6* and *Povp3* interact with lignin and cause its covalent bond to break? In our further study, this question will be investigated.

### Gene-relative transcript abundance profile of *PoPOD*s

**Fig. 5.**
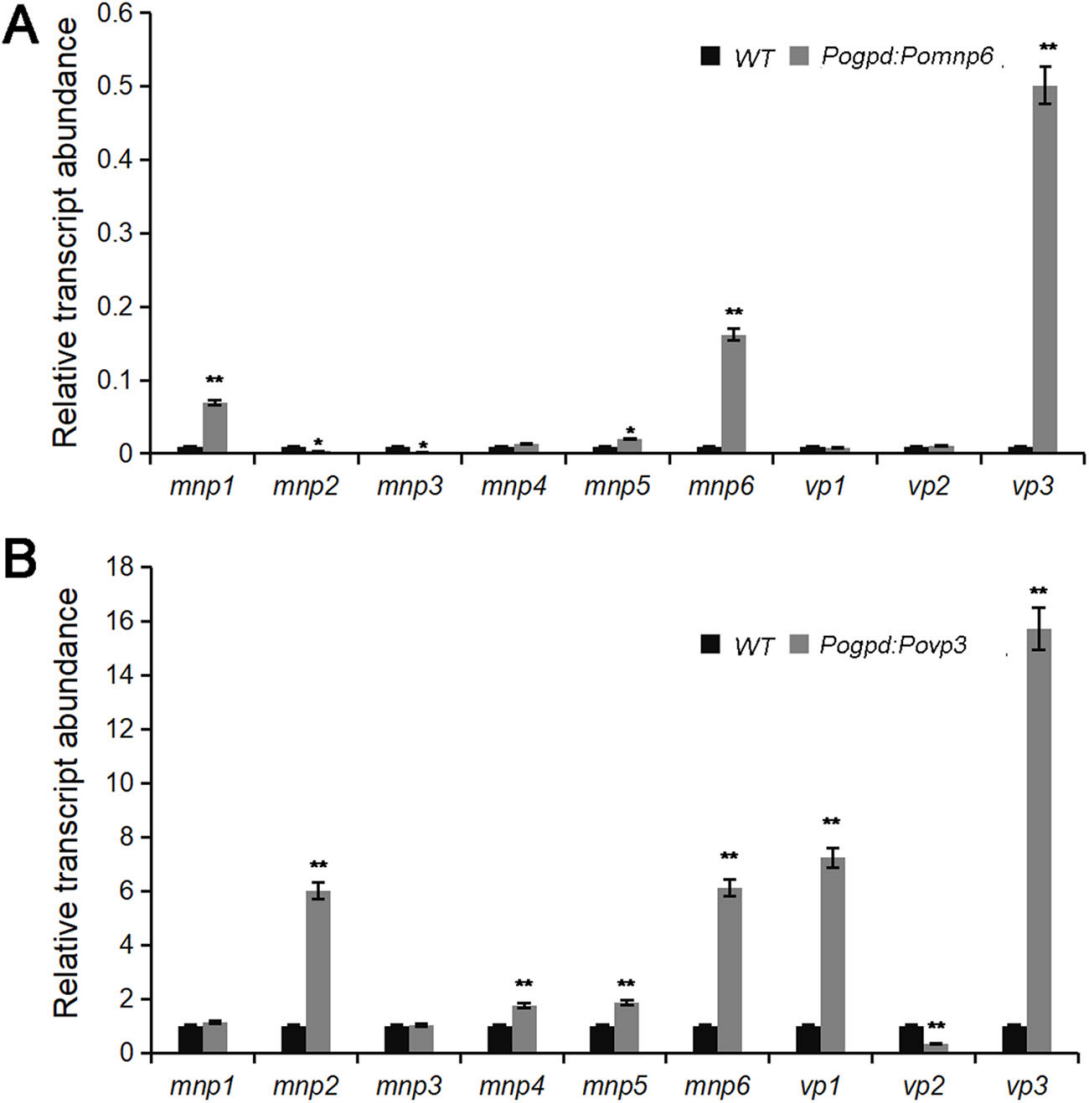
**Relative transcript abundance profile of *PoPOD*s after cultivating in the cotton solid medium for 20 days*.*** (A) Relative transcript abundance profile of *PoPOD*s after overexpression of *Pomnp6*. (B) Relative transcript abundance profile of *PoPOD*s after overexpression of *Povp3*. Each sample had three duplications. Values are mean±s.d. (*n*=3).

The PoPOD genes play a key role in the degradation of lignin. Therefore, the gene-relative transcript abundance profile of different *PoPOD*s in the transgenetic *P. ostreatus* was assayed (Fig. 5). As showed in Fig. 5A, the relative transcript abundance of *Pomnp1* and *Povp3* was upregulated in *Pomnp6* transformants compared with the wild-type strain (Fig. 5A). The relative transcript abundance of *Pomnp2*,* Pomnp6* and *Povp1* was drastically upregulated in contrast to other *PoPOD*s in *Povp3* transformants (Fig. 5B). These results indicated that degradation of cotton stalk lignin by *P. ostreatus* was a complex process; different lignin-degrading enzymes may play a synergistic role.

### Analysis of lignin structure by ^1^H-NMR

**Fig. 6.**
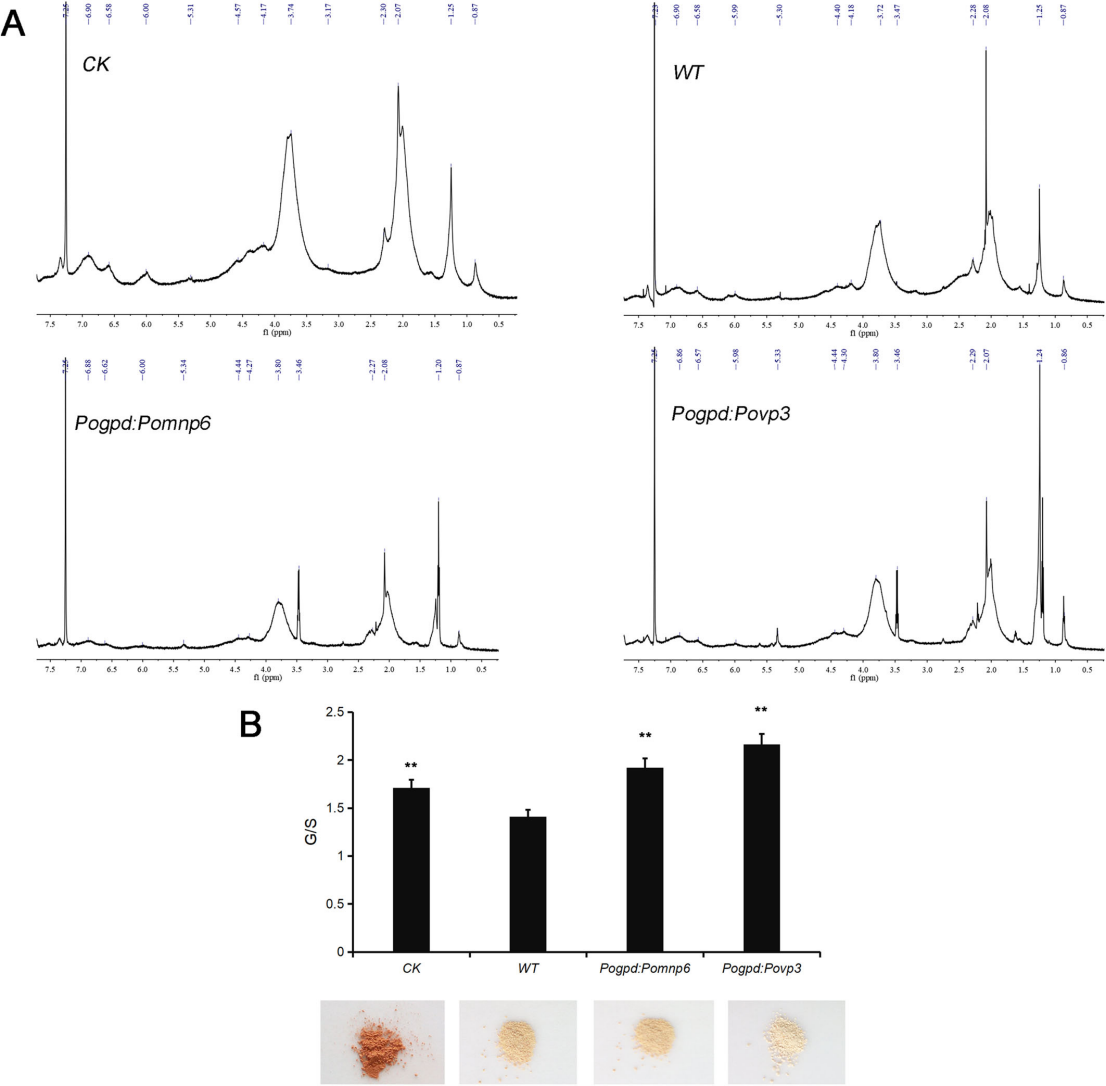
**^1^H-NMR spectra of acetylated MWL in cotton stalks.** (A) ^1^H-NMR spectra of acetylated MWL in cotton stalks. (B) The ratio of G/S showed in the histogram. CK, no fungus control. Each sample had two duplications. Student's *t*-test was used in this study. **P<0.01, *P<0.05.

^1^H-NMR analysis technology has been widely used as an effective method to determine the structure characteristics of lignin. To further investigate the changes in structure characteristics of cotton stalk lignin after *P. ostreatus* pre-treatment, milled wood lignin (MWL) was studied by ^1^H-NMR spectroscopy. The spectrum is shown in Fig. 6 and the characteristic signals of the cotton stalk lignin were shown in Table 1. The region of δ=7.2–6.7 were aromatic protons in guaiacyl units (G) and δ=6.7–6.3 were aromatic protons in syringyl units (S). The β-*O*-4′, β-5′ and β-β′ linkages were identified at δ=6.3–5.8, δ=5.2–5.6 and δ=4.3–4.0, respectively. In addition, H of methoxy groups and H of methyl were also detected in cotton stalk lignin from wild-type and transformant pre-treatments. To determine the relative contents of the various structures in the MWL, we integrated the signal intensities of the various sections of the hydrogen spectrum to characterize the proton numbers of each specific region. As shown in Table 2, MWL was G-S lignin and the G/S ratio was 1.71 in cotton stalks without fungal control. After culturing wild-type strains, the G/S ratio of the MWL was 1.41. The G/S ratio of the MWL was 1.92 (*Pogpd:Pomnp6*) and 2.17 (*Pogpd:Povp3*) after culturing transformants. Subsequently, the contents of β-*O*-4′, H of methoxy groups, H of aromatic acetates and H of aliphatic acetates were significantly decreased and β-5′ and β-β′ were slightly reduced after culturing transformants. Additionally, compared to the MWL treated with wild-type strains, the H of methylene was slightly increased and the H of methyl was reduced. The result indicated that *Pomnp6* and *Povp3* may preferentially degrade S-lignin in cotton stalks and the corresponding mainly break β-*O*-4′ bond linkages and hydroxyl. In order to further support this conclusion, the MWL was also studied by infrared spectrometer (FTIR). The results were basically consistent with the ^1^H-NMR conclusions, which further supported the rationality of the conclusion (Fig. S4, Tables S2 and S3).

**Table 1.**
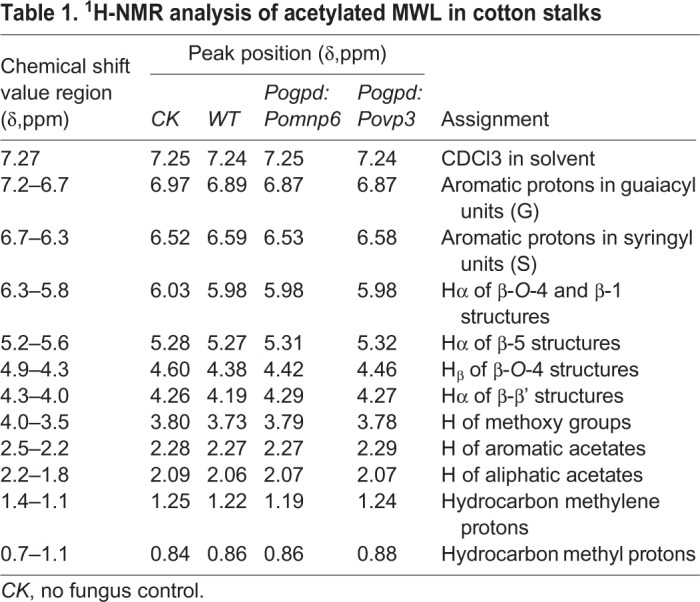
**^1^H-NMR analysis of acetylated MWL in cotton stalks**

**Table 2.**
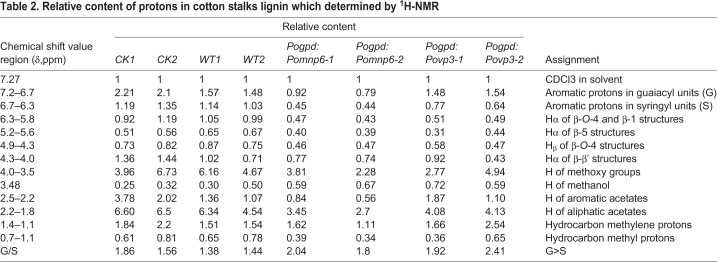
**Relative content of protons in cotton stalks lignin which determined by ^1^H-NMR**

## DISCUSSION

White-rot fungi heme peroxidases are a large group of biocatalysts with ecological and biotechnological significance. In this study, nine ligninolytic peroxidase genes were screened from *P. ostreatus*. Previous research had found that *Povp1* and *Povp2* had the function of lignin degradation (Fernández-Fueyo et al., 2014; Salame et al., 2014). According to the secretome of the model white-rot *P. ostreatus*, we inferred that *Pomnp3*, *Pomnp6* and *Povp3* may degrade lignin (Fernández-Fueyo et al., 2016). Combining evolutionary relation analysis (Fig. 1), gene structure (Fig. S1) and gene relative transcript abundance (Fig. 2), we finally selected *Pomnp6* and *Povp3* for further research. In addition, according to the phylogenetic tree, *P.* *eryngii* VPL1, VPL2 and VPL3 may also be involved in lignin degradation, which may provide some theoretical support for other experiments (Fig. 1).

Strong promoters are essential for establishing efficient gene expression and transformation systems. The *gpd* promoter has been proven to be one of strong and constitutive promoters in fungi (Shi et al., 2012). In this study, we cloned 1500 bp *gpd* promoters from the genome of *P. ostreatus.* Based on *ATMT*, we achieved the homologous overexpression of *Pomnp6* and *Povp3* in *P. ostreatus*, which significantly improved the lignin degradation of cotton stalks. *ATMT* system is widely used for plant species (Franklin and Lakshmi, 2003), and in recent years *ATMT* has been proved to be a powerful tool for transforming various fungal species such as *Vitreoscilla* and *Volvariella volvacea* (Wang et al., 2008; Zhang et al., 2017)*.* Our results also suggested that *ATMT* could be a reliable, feasible and promising technique for *P. ostreatus* transformation. In addition, we used *gus* as a reporter gene to analyze transient gene expression of *P. ostreatus*, which improved the detection efficiency. Compared to *gfp* and other reporter genes, it's easy, quick and efficient (Basu et al., 2003; Jefferson, 1987).

In this study, we have demonstrated that lignin degradation of cotton stalks mainly occurred in the mycelium stage (Fig. S2). Based on analysis of bioinformatics and overexpression, both *Pomnp6* and *Povp3* were found to be involved in enhancing the lignin biodegradation of cotton stalks (Fig. 4). To further clarify functional groups or chemical linkages within lignin polymers targeted by these two PODs, ^1^H-NMR spectrum analysis was performed and provided more detailed evidence that these two genes can degrade lignin in cotton stalks (Fig. 6). According to Table 2, the G/S ratio of the MWL after cultured transformants were higher than in wild type, so we indicated that *Pomnp6* and *Povp3* may preferentially degrade S-lignin in cotton stalks. Additionally, the relative contents of functional groups and linkages showed that *Pomnp6* and *Povp3* may mainly break β-*O*-4′ bond linkages and hydroxyl in cotton stalks lignin. The MWL data of FTIR further supported the rationality of this conclusion (Fig. S4 and Tables S2, S3). The work not only revealed the function of *Pomnp6* and *Povp3*, but also laid a foundation for better utilization of straw resources and provided the direction for the development of sustainable energy.

## MATERIALS AND METHODS

### Identification and characteristic of *PoPOD*s

Commercial dikaryon *P. ostreatus ‘*Suping1’ was provided by the Horticultural Institute of Anhui Academy of Agricultural Sciences. The genome data of Monokaryotic *P. ostreatus* PC15 (CECT20312) were obtained at the Joint Genome Institute (JGI) (http://genome.jgi.doe.gov/PleosPC15_2) (Alfaro et al., 2016; Castanera et al., 2016; Riley et al., 2014). Other basidiomycetes POD gene sequence data was obtained from National Center for Biotechnology Information (https://www.ncbi.nlm.nih.gov/).

### Phylogenetic tree construction

Multiple sequence alignments of nine PoPODs and another 27 basidiomycete PODs were generated using the ClustalW program. In the best model condition (G+I), a phylogenetic tree was constructed using MEGA 5.1 with maximum likelihood method (bootstrap=1000) (Zhou et al., 2018). Subsequently, exon-intron structures were generated using GSDS (http://gsds.cbi.pku.edu.cn/) and conserved motifs were confirmed using Multiple Em for Motif Elicitation (MEME) (http://meme-suite.org/) (Bailey et al., 2009; Hu et al., 2015)*.* The maximum value of the motif was set to 20, and the length of the sequence was set between 6∼200. In addition, Pfam (http://pfam.xfam.org/) was used to further analyze each motif (Su et al., 2017).

### Determination of lignin content and MnP activity

*P. ostreatus* was cultured on the cotton medium containing 5 g cotton stalk powder (with particle size less than 0.25 mm) and 22 ml of liquid medium. The latter was made up of ammonium tartrate (22.0 g/l), macroelements (KH_2_PO_4_ 20 g/l, MgSO_4_·7H_2_O 13.8 g/l, CaCl_2_ 1.0 g/l, NaCl 0.6 g/l), microelement (MnSO_4_·H_2_O 0.35 g/l, FeSO_4_·7H_2_O 60 mg/l, CoCl_2_·6H_2_O 110 mg/l, ZnSO_4_·7H_2_O 60 mg/l, CuSO_4_·5H_2_O 95 mg/l, AlK(SO_4_)2·12H_2_O 6 mg/l, H_3_BO_3_ 6 mg/l, Na_2_MoO_4_·2H_2_O 6 mg/l), VB_1_ (100 mg/l) and water in a certain proportion of 1:15:15:3:16. After sterilization, *P. ostreatus* was inoculated into each bottle and cultured in 28°C incubators for 10 days, 15 days, 20 days, 25 days, 30 days, 35 days, 40 days, 50 days and 60 days, respectively.

The mycelium was cultured in the absence of light for the first 30 days and the fruiting stage was followed 30 days. Finally, mycelia and fruit body samples of different periods were taken and rapidly frozen using liquid nitrogen for qRT-PCR analysis. Each bottle of the culture medium was filled with 15 ml of pure water (12 h at 4°C), followed by extraction for 1 h (4°C, 180 r/min) and the leachate was centrifuged at 6000 rpm for 10 min at 4°C. The supernatant was used to measure MnP activity. The solid residue substrate was oven-dried at 60×C to a stable weight, and 1 g was accurately weighed by measuring the lignin content using a Hanon F800 fiber analyzer (Jinan, China). All of them were set up as three duplicate groups.

The MnP activity was measured by oxidizing 1.6 mM MnSO_4_ (ɛ=8100 M^−1^ cm^−1^) at 240 nm within 3 min. The reaction mixture was 3.4 ml of 50 mM sodium acetate buffer (pH 4.5), 0.1 ml of 1.6 mM MnSO_4_, 0.4 ml of enzymes, and then 0.1 ml of 1.6 mM H_2_O_2_ was added to start the reaction. One unit of enzyme activity is defined as the enzymes converting 1 μmol of Mn^2+^ to Mn^3+^ in 1 min (Praveen et al., 2012).

### RNA extraction and quantitative real-time PCR

The *PoPOD* sequences were obtained from *P. ostreatus* genome, qRT-PCR primers were designed (Table S4) and were synthesized by Sangon Biotech Co., Ltd. (Shanghai, China). The RNAprep Pure Plant Kit (Tiangen, China) was used to extract total RNA from *P. ostreatus* samples. Additionally, reverse transcription was performed using TransScript® One-Step RT-PCR SuperMix (Trans, China) and qRT-PCR was performed using the Bio-rad Cfx96 Touch™ Deep Well Real-Time PCR detection system. The PCR volume was 20 μl, and *Cyph* (transcript ID: 1058252) was set as the reference gene (Castanera et al., 2012). Three parallel replicates were set for each sample. Finally, relative gene expression was calculated by the 2^−△△Ct^ method (Livak and Schmittgen, 2001). The reactions were contained in the following: 10 μl of TransStart Tip Green qRT-PCR SuperMix (2×) (Trans, China), 2 μl of template cDNA, 0.8 μl of forward and reverse primers and ddH_2_O to 20 μl. The PCR amplification conditions were performed as follows: 98°C for 2 min, followed by 40 cycles of 98°C for 10 s, 60°C for 10 s and 68°C for 30 s.

### Gene cloning and vector construction

The *glyceraldehyde-3-phosphate dehydrogenase* (*gpd*) promoter (GenBank accession no. KL198006.1) was cloned from *P. ostreatus*. Then it was inserted into the expression plasmids pCAMBIA1304 (GenBank: AF234300.1) to replace its *CAMV35S* promoter by using the restriction sites of HindIII and NcoI*.* Subsequently, *Pomnp6* (GenBank accession no. MF681783) and *Povp3* (GenBank accession no. MF681784) were cloned from *P. ostreatus* using specific primers (Table S5), respectively. All amplified PCR products were purified, sub-cloned with the pMD18-T vector (Takara, China) and sequenced (Sangon, China). The PCR products were digested with BglII and SpeI (Takara), then inserted into the pCAMBIA1304-*Pogpd* vetor (Sharma and Kuhad, 2010). Final vector plasmids were designated as pCAMBIA1304-*Pogpd-Pomnp6* and pCAMBIA1304-*Pogpd-Povp3.*

### ATMT of *P. ostreatus*

The *A. tumefaciens* strains EHA105, harboring pCAMBIA1304-*Pogpd-Povp3* and pCAMBIA1304-*Pogpd-Pomnp6*, respectively, were cultivated at 28°C in LB medium (containing antibiotics kanamycin and rifampicin) to an OD_600_ of 0.6-0.8. Bacterial cells were then collected, centrifugated, and suspended in an induction medium (IM, including 200 μmol/l acetosyringone) to an OD_600_ of 0.5-0.6, pH 5.5, and the virulence of *A. tumefaciens* was induced by shaking at 150 rpm for 6 h at 28°C (Zhang et al., 2015)*.*

The fungal mycelia were grown on PDA (potato 200 g/l, dextrose 20 g/l, agar 20 g/l) for 7 days *P. ostreatus* mycelium (0.9×0.9 cm) was immersed in pre-inducing bacterial culture for 30 min and then placed on solid induction medium (IM, including 200 μmol/l acetosyringone) for 4 days. Subsequently, the co-cultured mycelia were transferred to a selection agar medium (SM) containing 100 μg/ml hygromycin and 300 μg/ml cefotaxime. The stability of the transformation was confirmed by subculturing colonies onto a fresh selective medium three times, then cultured on PDA for three generates to rejuvenate mycelia.

### PCR analysis and visual detection of β-glucuronidase (GUS)

The genomic DNA of putative transformants was extracted using the EasyPure®Plant Genomic DNA Kit (Trans). In order to confirm that the gene has been integrated into the genome, PCR analysis was performed. The primers gusF and gusR (Table S2) for amplification of the *gus* were designed to verify whether the T-DNA was inserted. The PCR conditions were set as follows: 94°C for 3 min, followed by 35 cycles of amplification 94°C for 30 s, 59°C for 30 s, 72°C for 100 s and ended after 72°C for 10 min. In order to verify the expression of the introduced *gus* reporter gene, putative transformants were detected by GUS histochemical assay kit (Real-Times, China).

### Extraction and purification of milled wood lignin (MWL)

After cultivating for 30 days with the wild type and transformants, cotton stalks were removed and dried at 40°C. According to the previous methods (Yan et al., 2014), MWL was extracted and purified. With a powder/extraction ratio of 1:10, the powder was extracted with a reflux mixture (dioxane/water, 8:2) at 25°C for 36 h. The raw lignin was obtained after the solvent was removed by rotary evaporation under vacuum at 40°C. The raw lignin was completely dissolved in a mixture of pyridine, glacial acetic acid and water (volumetric ratio of 9:1:4), combined with trichloromethane and separated using a separatory funnel. The chloroform layer (bottom layer) was collected and mixed with ether to form a precipitate. The precipitate was isolated by centrifugation and washed repeatedly with diethyl ether until no pyridine odor was observed. Then after drying in a vacuum oven at 40°C, the resulting material is purified MWL.

### Infrared spectrometer (FTIR) of MWL

The MWL (2.0 mg) in cotton stalks was mixed with 100 g KBr in a dry environment, ground uniformly into a powder and pressed into thin slices. Infrared spectrometer Nicolette is50 was scanned 32 times in the range of 500–5000 cm^−1^ per material and averaged. The resolution and accuracy of wavenumbers were 4 cm^−1^ and 0.01 wave numbers. The A1271/A1223 ratio represented the proportion of G-lignin and S-lignin (Yan et al., 2014).

### ^1^H-NMR

Lignin (50.0 mg) was dissolved in a 2.0 ml mixture of pyridine and acetic anhydride (1:1). The flask was filled with nitrogen gas and incubated at 25°C in the dark for 72 h. The acetylated MWL was then precipitated by adding ether, centrifuged at 5000 rpm and washed until the odor of pyridine was absent. This material was dissolved in 0.5 ml of CDCl_3_ and analyzed with an Agilent DD2 NMR spectrometer at 600 Hz. The tetramethylsilicone (TMS) as the internal standard. The relative content of protons in the aromatic acetyl group and the aliphatic acetyl group is equivalent to the relative content of the alcoholic hydroxyl group and the phenolic hydroxyl group. The ratio of guaiacyl and syringa substrates represents the ratio of G-lignin to S-lignin (G/S) (Cai et al., 2010).

## Supplementary Material

Supplementary information
