# Correction: Molecular characterization and overexpression of *mnp6* and *vp3* from *Pleurotus ostreatus* revealed their involvement in biodegradation of cotton stalk lignin

**DOI:** 10.1242/bio.059735

**Published:** 2023-01-17

**Authors:** Yan Wang, Guoqing Li, Xiaoyu Jiao, Xi Cheng, Muhammad Abdullah, Dahui Li, Yi Lin, Yongping Cai, Fan Nie

There was an error published in *Biol. Open* (2019) **8**, bio036483 (doi:10.1242/bio.036483).

In Fig. 6, the *Pogpd:Pomnp6* image in panel B was erroneously a duplication of the WT image.

The corrected and original figures are shown below. Both the online full-text and PDF versions of the article have been updated. The authors apologise to readers for this error, which does not impact the results or conclusions of this paper.

**Fig. 6 (corrected).**
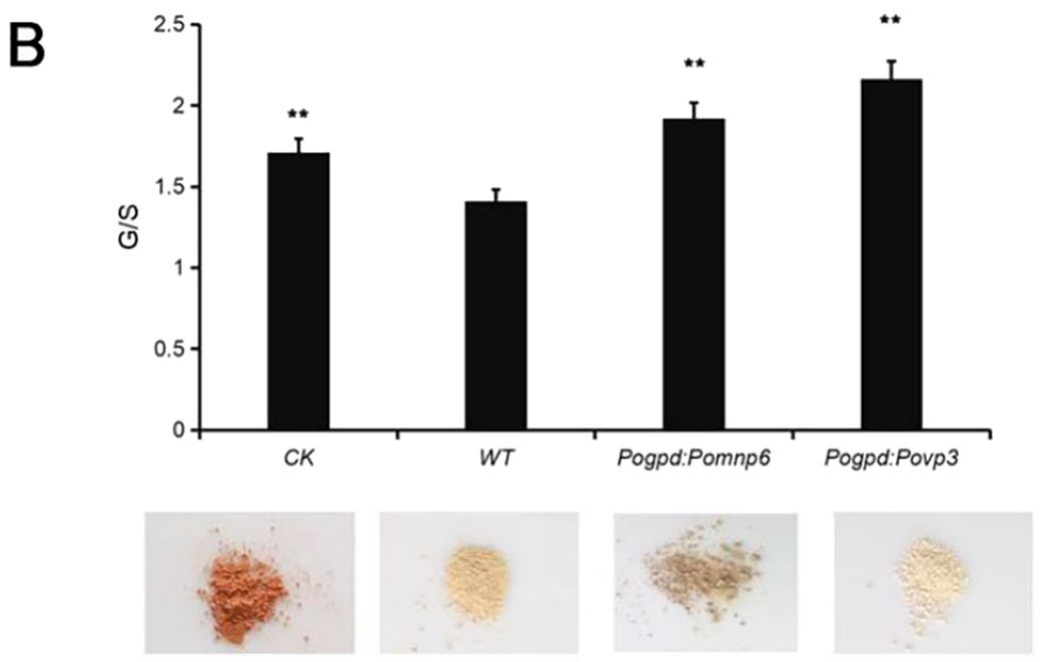
**^1^H-NMR spectra of acetylated MWL in cotton stalks.** (B) The ratio of G/S showed in the histogram. CK, no fungus control. Each sample had two duplications. Student's *t*-test was used in this study. **P<0.01, *P<0.05.

**Fig. 6 (original).**
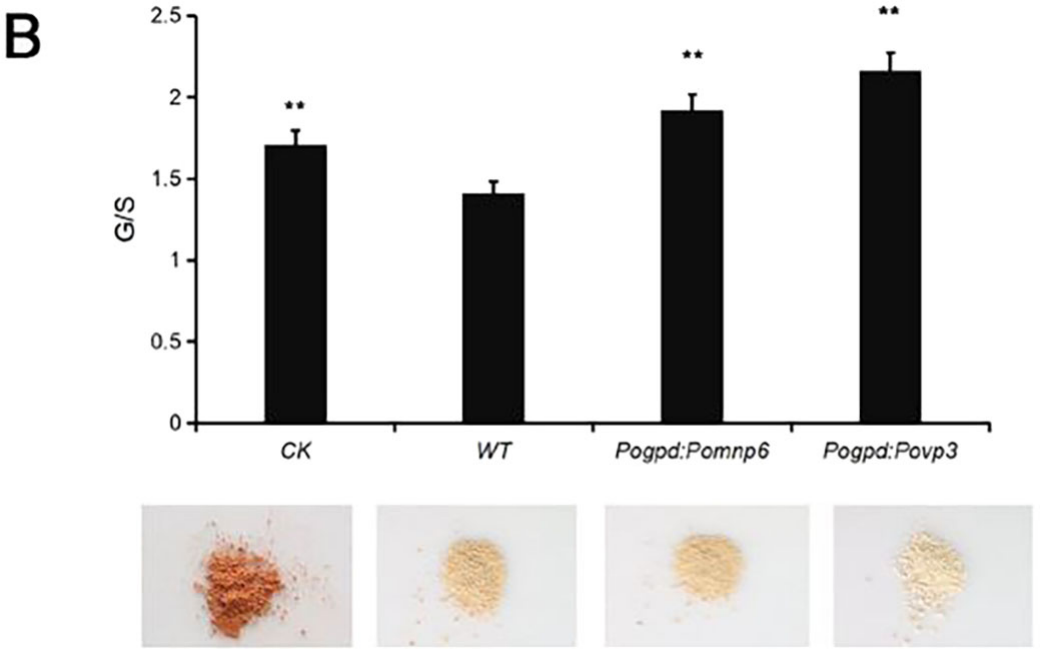
**^1^H-NMR spectra of acetylated MWL in cotton stalks.** (B) The ratio of G/S showed in the histogram. CK, no fungus control. Each sample had two duplications. Student's *t*-test was used in this study. **P<0.01, *P<0.05.

