# Supplementary material for: Molecular characterization and overexpression of mnp6 and vp3 from Pleurotus ostreatus revealed their involvement in biodegradation of cotton stalk lignin
Source: Biol Open. 2018 Dec 24;8(2):bio036483. doi: 10.1242/bio.036483 (PMC6398461; doi:10.1242/bio.036483)
Supplement: Supplementary information [file biolopen-8-036483-s1.pdf]

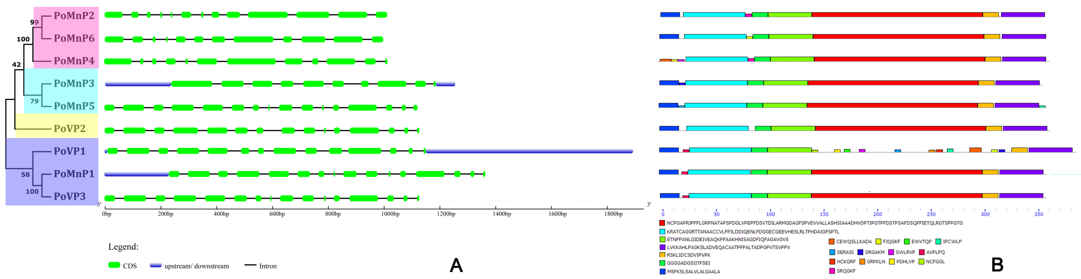

Fig. S1. Gene structures and conserved motifs analysis of PoPODs. (A) Exon/intron structure map of PoPODs. Exons, introns and UTRs were indicated by green boxes, black lines and blue boxes, respectively. Relative protein or gene lengths were estimated by rulers. (B) Protein conserved motifs of PoPODs. Different color boxes represented different motifs.

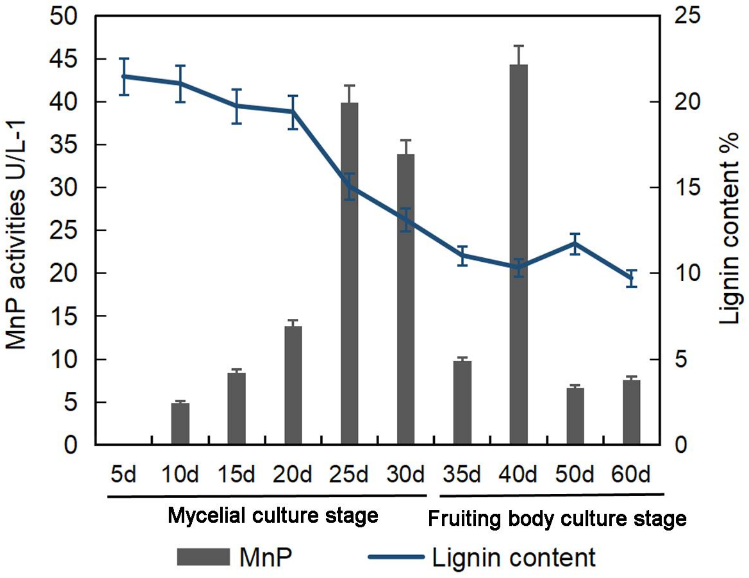

Fig. S2. Lignin content of cotton stalks and *P. ostreatus* MnP activities in different periods. The *P. ostreatus* grown on cotton solid medium for 10 d, 15 d, 20 d, 25 d, 30 d, 35 d, 40 d, 50 d and 60 d. The previous thirty days were mycelium stages, and later thirty days were fruiting stages.

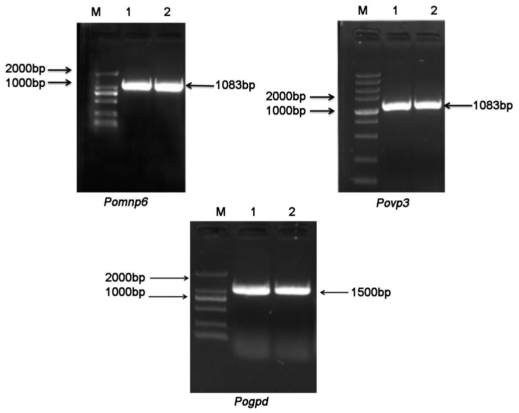

Fig. S3. Amplification of *Pogpd*, *Pomnp6* and *Povp3* fragments.

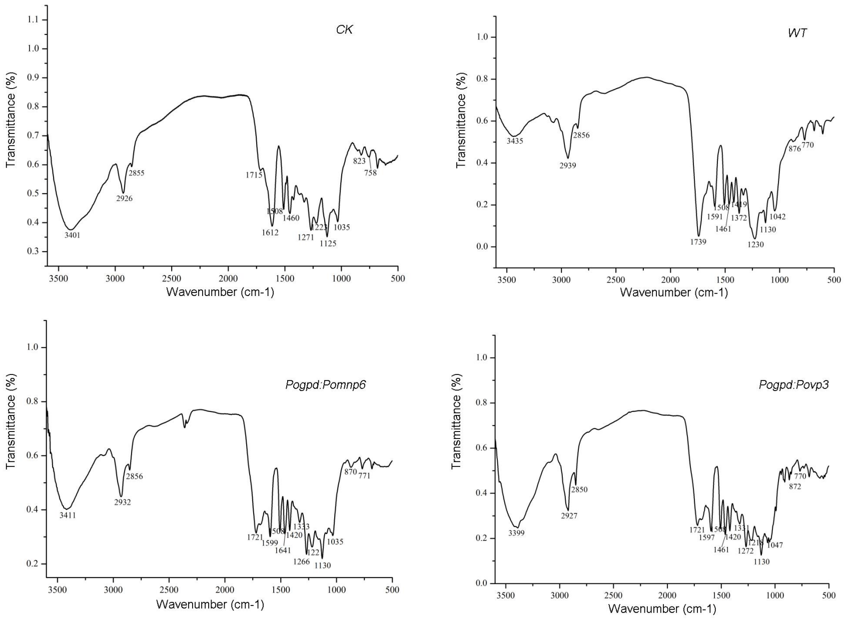

Fig. S4. FTIR analysis of MWL lignin in cotton stalks.

Biology Open • Supplementary information

Table S1. PoPODs main motifs information

Motif	Amino acid numbers	Conserved amino acid sequences	Feature domain
Motif 1	159	NCPGAPRIYYYLGRPNYTYPSPDYLVPEPYDSVTYI LARMGDAGYYPYEVVWLLASHTIAAADYVDETIP GTPYDSTPSYYDSQYYIETQLRGTYYPGYGGNYGE VESPLYGEMRLQSDYLLARDYRTACEWQSYVNNQ YKMQNRYYYYMYKMSYLGQN	Peroxidase
Motif 2	58	KRATCAGGRTTANAACCVLFPILDDIQENLFDGGE CGEEVHESLRLTFHDAIGFSPTL	*
Motif 3	41	ETNFPANLGIDEIVEAQKPFAAKHNISAGDFIQFAGA VGVS	*
Motif 4	41	LVKKAHLPAGKSLADVEQACAATPFPALTADPGPV TSVPPV	Fungal peroxidase extension region
Motif 5	15	RSKLIDCSDVIPVPK	*
Motif 6	15	GGGGADGSIITFSEI	*
Motif 7	18	MSFKSLSALVLALGAALA	*
Motif 8	6	SRQGKF	*
Motif 9	11	CEWQSLLKADA	*
Motif 10	6	FJQGKF	*
Motif 11	6	EWVTQP	*
Motif 12	6	IPCWLP	*
Motif 13	6	SERASI	*
Motif 14	6	GRGAKM	*
Motif 15	6	SWLRVP	*
Motif 16	6	AVPLPQ	*
Motif 17	6	HCKGRF	*
Motif 18	6	GRPKLN	*
Motif 19	6	PDHLVP	*
Motif 20	6	NCFGGL	*

* represents there is no feature domain.

Biology Open • Supplementary information

Table S2. The assignment of FTIR absorption peak positions and changes in the lignin structure of the cotton stalks

Absorption band (cm^{-1})				Assignment
CK	*WT*	*Pogpd: Pomnp6*	*Pogpd: Povp3*	
3401	3435	3411	3399	O-H stretching vibrations in aromatic and aliphatic
2926	2939	2932	2927	C-H stretching vibrations in methyl and methylene groups
2855	2856	2856	2850	C-H vibrations in methoxyl groups
1715	1739	1721	1721	C=O vibrations in unconjugated carbonyl groups
1612	1591	1599	1597	Aromatic skeletal vibrations plus C=O vibration
1508	1508	1508	1508	Aromatic skeletal vibrations
1460	1461	1461	1461	C-H deformations band of asymmetric methyl and methylene
1372	1372	1372	1372	C-H stretching vibrations in aliphatic plus O-H out of plane bending vibration
1325	1331	1333	1331	Syringyl units
1271	1272	1266	1272	Guaiacyl units
1223	1230	1221	1218	Aromatic C-O stretching vibrations (S units)
1126	1130	1130	1130	Aromatic C-H in plane deformations
1035	1042	1035	1047	Aromatic C-H in plane deformation plus C-O deform in primary alcohols plus C=O stretching
858	876	870	872	Aromatic C–H out of plane bending vibration

Table S3. The ratio intensities of G-lignin and S-lignin in cotton stalks as determined by FTIR

	CK	*WT*	*Pogpd:Pomnp6*	*Pogpd:Povp3*
A1271/A1508	1.2155	1.3638	1.2715	1.2965
A1223/A1508	1.1467	2.0571	1.1773	1.1949
A1271/A1223	1.0600	0.6630	1.0799	1.0851

Biology Open • Supplementary information

Table S4. Primer sequences used for qRT-PCR

Gene	Transcript identification no. (PC15)	Primer name	Primer sequence (5'-3')
mnp1	1096331	Fw	CAGTCAGATCACTTGTTC
		Rv	ATCTTCTGTTGGTCGTTA
mnp2	199510	Fw	GACATTCAAGAGAACCTATT
		Rv	AGAATCCGATAGCATCAT
mnp3	1089546	Fw	CTTCAATCCGCTTTCAAG
		Rv	ATAACATCAGAGCAGTCAA
mnp4	1099081	Fw	ATTCGCAGTTCTTCGTTGA
		Rv	ACTTGACTTGACCCTTGTG
mnp5	199511	Fw	GATGGCAACACTGTCACTA
		Rv	GAAGAGGTTGGTCTGGATG
mnp6	1041740	Fw	AATGGCAATCCTTCATCA
		Rv	CCTAGAGTGGACATCTTG
vp1	1089895	Fw	CTCTCCGCTCTTGTGCTTG
		Rv	GGGAACAGAACGCAACATG
vp2	199491	Fw	GAAGATCCAGAACAGATT
		Rv	AATAACATCAGAGCAGTC
vp3	156336	Fw	GGCTAACCTCGGTATTGAC
		Rv	TCACCTGCGGATATGTTG
cyph	1058252	Fw	GACATTGCTATCGACTCCCAG
		Rv	GAAATTCCTTGCAGTCTTGGG

Table S5. Primer sequences used for RT-PCR

Primer name	Primer sequence (5'-3')
gpd-F	CCCAAGCTTTCGAGGCTACCTCGCTACTG
gpd-R	CATGCCATGGTTCAAGGCCGTTGTATTAGT
mnp6-F	GAAGATCTGATGTCTTTCAAGGCTCTATTCACTT
mnp6-R	GGACTAGTCACAGGAGGAACGGTGGT
vp3-F	GAAGATCTGATGACCTTCGCCTCTCTTTCC
vp3-R	GGACTAGTCGAAGGGGGGACGGG
gus-F	GTCCTGTAGAAACCCCAACCCGTGA
gus-R	TTTGCCTCCCTGCTGCGGTTTTTCA

Table S6. Gene amplification efficiency of *P. ostreatus PODs*

Gene name	Efficiency	R^2
cyph	103.0%	0.995
mnp1	91.3%	0.996
mnp2	98.1%	0.971
mnp3	104.5%	0.997
mnp4	99.4%	0.998
mnp5	100.9%	0.997
mnp6	107.3%	0.989
vp1	98.6%	0.987
vp2	97.7%	0.999
vp3	93.0%	0.994

cyph

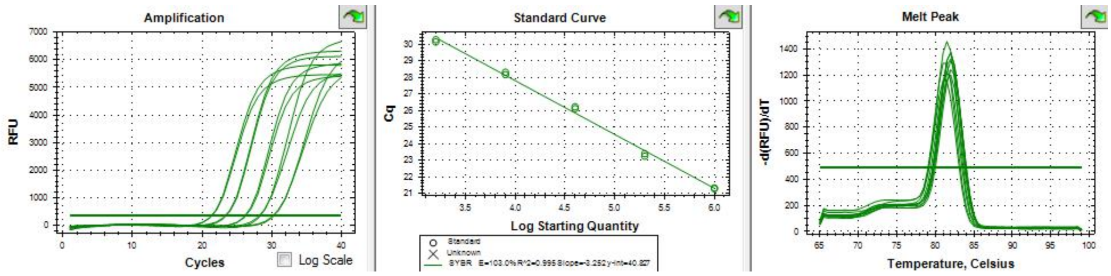

mnp1

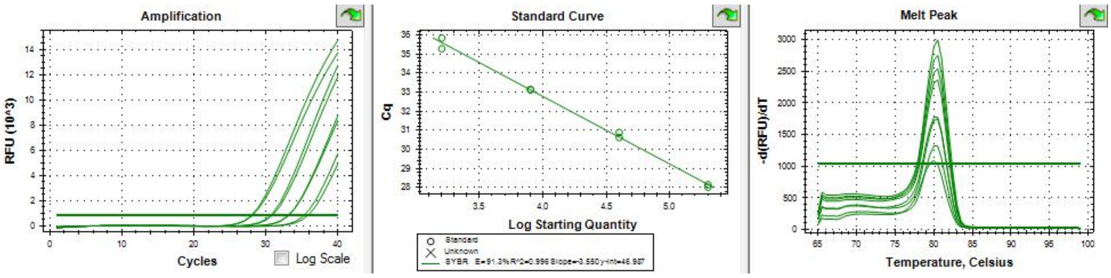

mnp2

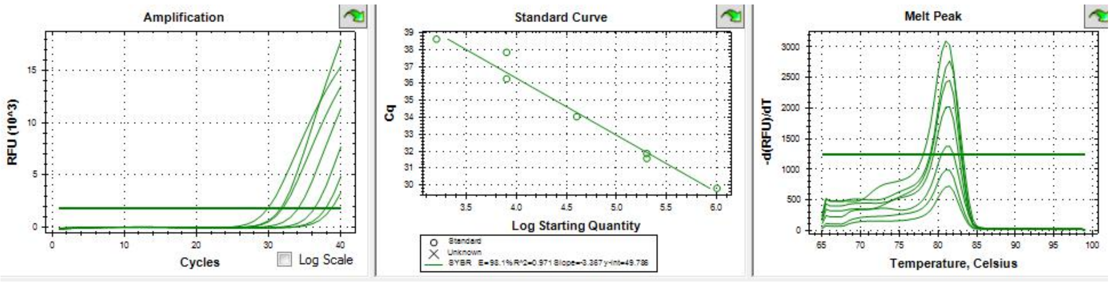

Biology Open • Supplementary information

mnp3

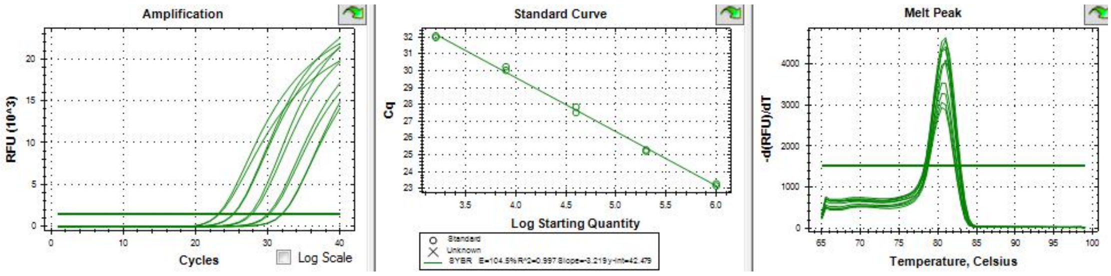

mnp4

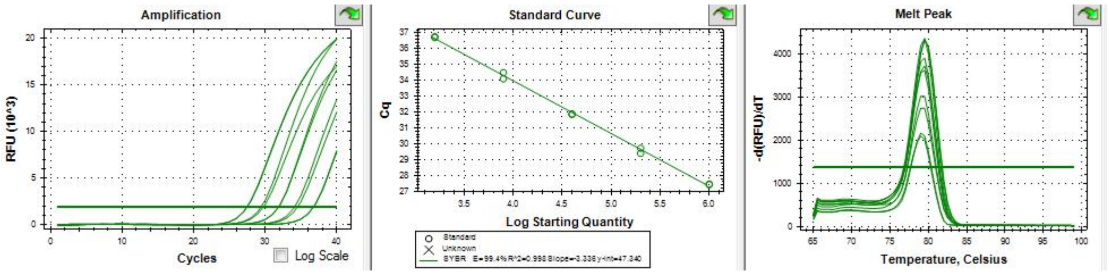

mnp5

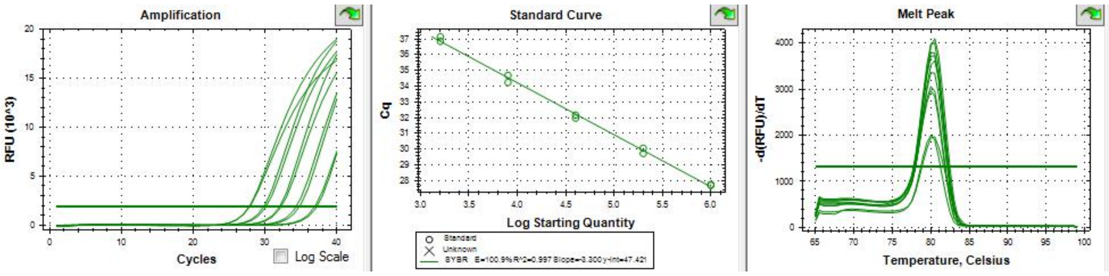

mnp6

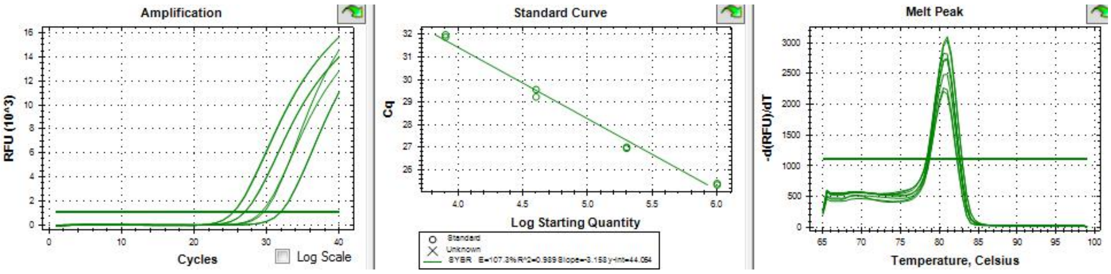

vp1

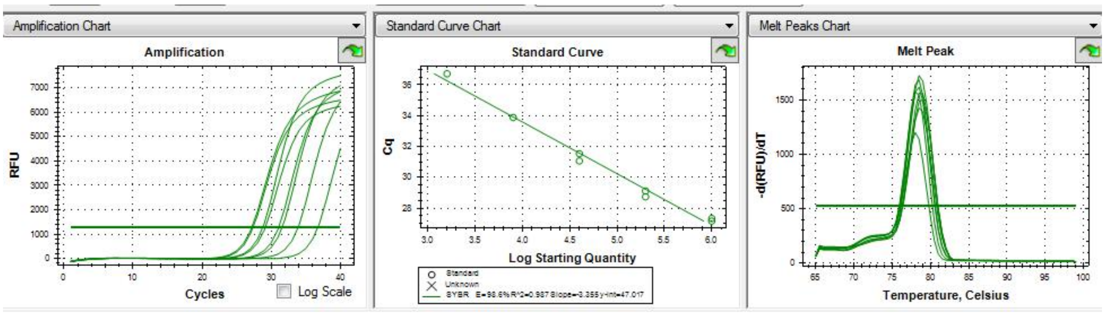

Biology Open • Supplementary information

vp2

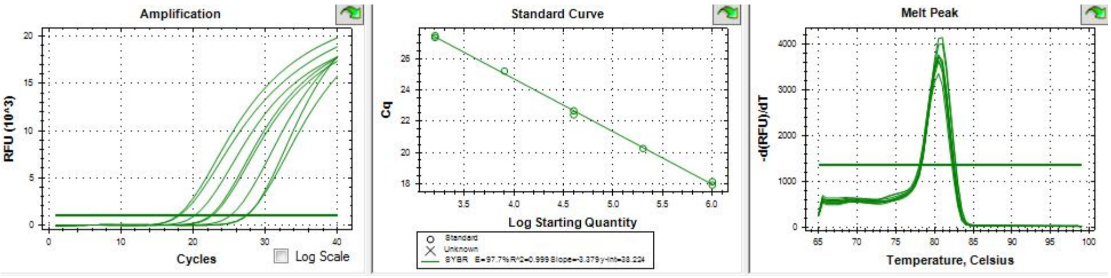

vp3

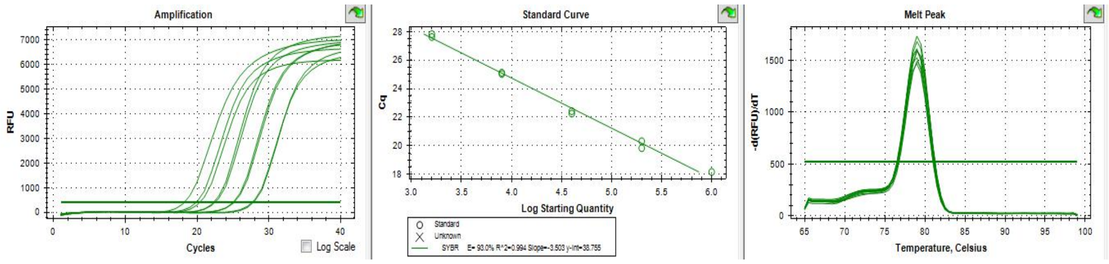

Biology Open • Supplementary information
